# Design, Synthesis and Cytotoxicity of Novel Dihydroartemisinin-Coumarin Hybrids via Click Chemistry

**DOI:** 10.3390/molecules21060758

**Published:** 2016-06-10

**Authors:** Ye Tian, Zhen Liang, Hang Xu, Yanhua Mou, Chun Guo

**Affiliations:** 1 Key Laboratory of Structure-Based Drug Design and Discovery of Ministry of Education, School of Pharmaceutical Engineering, Shenyang Pharmaceutical University, Shenyang 110016, China; tianye870918@126.com (Y.T.); llzz_666@126.com (Z.L.); xuhanglg2@126.com (H.X.); 2 Department of Pharmacology, Shenyang Pharmaceutical University, Shenyang 110016, China; mu_hua_jj@sina.com

**Keywords:** anticancer, artemisinin, coumarin, hybrid, carbonic anhydrase IX, cytotoxicity, anoxic

## Abstract

In order to develop novel chemotherapeutic agents with potent anticancer activities, we designed four series of novel compounds employing hybridization strategy. Twenty novel dihydroartemisinin-coumarin hybrids, **10a**–**e**, **11a**–**e**, **12a**–**e**, **13a**–**e**, were synthesized via click chemistry in this study and their structures were characterized by HRMS and NMR. The cytotoxic activities were measured by MTT assay against three cancer cell lines (HCT-116, MDA-MB-231, and HT-29) under normoxic or anoxic conditions, respectively. The target compounds exhibited moderate activity with IC_50_ values in the 0.05–125.40 μM range, and these compounds exhibited better activity against HT-29 cell line under anoxic condition. The cytotoxic activities of most compounds under anoxic condition displayed one- to 10-fold greater activity than under normoxic condition. Compounds **10a**–**e** showed better selectivity against the HT-29 cell line than the other two cell lines. These results indicated that our design of CA IX inhibitors does correspond with its action mode to some degree and deserves further investigation.

## 1. Introduction

Currently, the mortality rate associated with cancer shows an increasing tendency worldwide. Chemotherapy is the most important therapeutic strategy against all types of cancer; however, the available anticancer drugs usually cause adverse effects and lead to the development of resistance. Therefore, there is a pressing need for novel chemotherapeutic agents with higher selectivity and more potent anticancer activities [1,2].

Many natural products have been found to possess antineoplastic activity with minimal side effects [3]. Artemisinin, a well-known anti-malarial agent, has been reported to possess a potent and broad antitumor spectrum against human cancer cell lines [4]. However, the drawback, such as drug resistance or unsatisfactory metabolism, constrains artemisinin to be employed as an antitumor drug clinically. Numbers of artemisinin derivatives have been designed and synthesized to overcome the imperfection mentioned above and some of them have shown excellent anticancer activities and pharmacokinetic properties [5].

Carbonic anhydrases (CAs, EC 4.2.1.1) are ubiquitous zinc metalloenzymes [6] that catalyze a very simple, but important, reaction: the reversible hydration of carbon dioxide to bicarbonate ion and proton, following a two-step catalytic mechanism [7]. Out of the sixteen human CA (hCA) isozymes that have been identified, CA IX is a tumor-associated isoform which over-expressed in a broad range of solid tumor types, while in normal tissues, the expression was much more limited, mainly in the glandular mucosa region in stomach [8,9]. In anoxia environments, the expression of CA IX is important in tumor growth and metastasis by increasing the tumor cell survival and invasion, consequently, CA IX is considered to be a promising anti-cancer drug target [10,11,12] and developing high selective inhibitors of CA IX may reduce side effects compared to classical anticancer agents [13]. In the last years, several approaches have been reported that the coumarins are truly CA IX-selective inhibitors [14,15,16,17] which exhibit a very different binding mode [17].

**Scheme 1:**
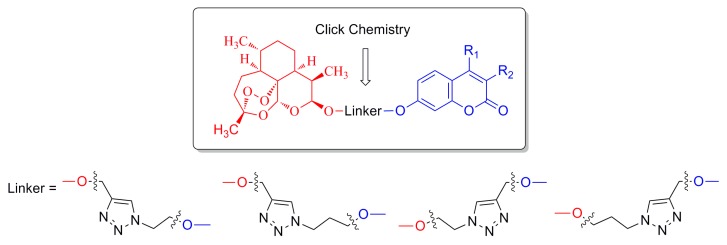
The design of target compounds.

Using the principle of hybridization, we designed and synthesized four new series of artemisinin derivatives by means of click chemistry (Scheme 1), in which the coumarin moiety was attached to C-10 position of dihydroartemisinin to afford new hybrids. The details of the synthesis of target compounds and evaluation of their activities against cancer cell lines are presented herein. 

## 2. Results and Discussion

### 2.1. Chemistry

**Scheme 2:**
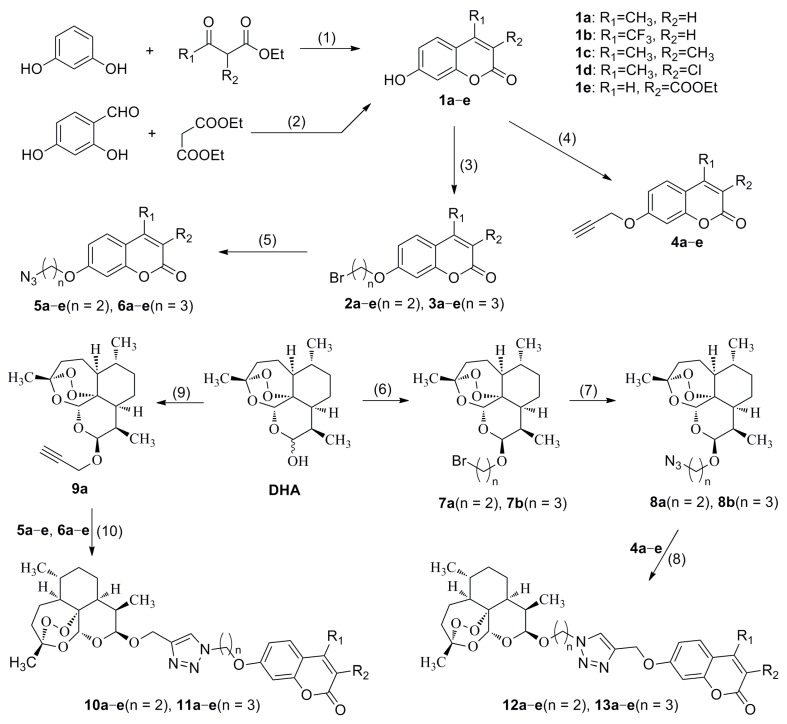
Synthetic routes to target compounds **10a**–**e**, **11a**–**e**, **12a**–**e** and **13a**–**e**. *Reagents and conditions*: (1) H_2_SO_4_/r.t. 12 h; (2) piperidine/EtOH/reflux 8–10 h; (3) 1,2-dibromoethane or 1,3-dibromoproane/Me_2_CO/K_2_CO_3_/56 °C 10 h; (4) 3-bromo-1-propyne/DMF/K_2_CO_3_/60 °C 4–5 h; (5) NaN_3_/ DMF/70 °C 4–5 h; (6) 2-bromoethanol or 3-bromo-1-propanol/BF_3_·Et_2_O/CH_2_Cl_2_/0 °C 6–8 h; (7) NaN_3_/DMF/70 °C 4–5 h; (8) CuSO_4_·5H_2_O/sodium ascorbate/DMF/r.t. 8–10 h. (9) 2-propyn-1-ol/BF_3_·Et_2_O/CH_2_Cl_2_/0 °C 6–8 h; (10) CuSO_4_·5H_2_O/sodium ascorbate/DMF/r.t. 8–10 h.

The synthetic approaches toward the target compounds **10a**–**e**, **11a**–**e**, **12a**–**e**, and **13a**–**e** were illustrated in Scheme 2, proceeded in five steps, commencing from 1,3-dihydroxybenzene or 2,4-dihydroxybenzaldehyde, and DHA.

The coumarin derivatives **1a**–**e** were synthesized according to the literature [18,19]. Reaction of 1,3-dihydroxybenzene with corresponding substituted ethyl acetoacetate in H_2_SO_4_ afforded substituted coumarins (**1a**–**d**) [18], while **1e** was synthesized by 2,4-dihydroxybenzaldehyde cyclized with diethyl malonate [19]. The substituted coumarins (**1a**–**e**) were then reacted with 1,2-dibromoethane, 1,3-dibromopropane or 3-bromo-1-propyne, respectively, to give **2a**–**e**, **3a**–**e**, **4a**–**e**, **7a**, **7b**, and **9a** were obtained by dihydroartemisinin(DHA) reacted with 2-bromoethanol, 3-bromo-1-propanol, or 3-bromo-1-propyne, respectively, as β configuration (indicated by the small coupling constants between 9-H and 10-H in ^1^H NMR, *J*_9,10_ = 3–4 Hz [20]) by using boron trifluoride ethyl ether as a catalyst [21]. Further azidation of **2a**–**e**, **3a**–**e**, **7a**, and **7b** gave compounds **5a**–**e**, **6a**–**e**, **8a**, and **8b** [22].

Finally, the intermediate bearing azido group (**5a**–**e**, **6a**–**e**, **8a**, **8b**) reacted with the intermediate possessing terminal alkyne group (**4a**–**e**, **9a**), respectively, (**5a**–**e**, **6a**–**e** with **9a**; **8a**, **8b**, with **4a**–**e**) via click chemistry [23] to get the target compounds.

### 2.2. Cytotoxicity

We focused our attention on the cytotoxicity of the four series of newly-synthesized compounds (**10a**–**e**, **11a**–**e**, **12a**–**e**, and **13a**–**e**) under normoxic or anoxic conditions. The cytotoxic activities of target compounds were measured by MTT assay, working with MRC-5 (human fetal lung fibroblast cells, normal lung cell), HCT-116 (human colorectal cancer cell line, do not express CA IX in response to anoxia), MDA-MB-231 (human breast carcinoma cell line, CA IX negative), and HT-29 (human colon carcinoma cell line, overexpresses high amounts of CA IX) [24,25], using doxorubicin as a positive control, and acetazolamide (AAZ, standard carbonic anhydrase inhibitor) as reference drug.

The data was calculated by the Logit method and presented as IC_50_ values in Table 1. In 96 h MTT assay results, cytotoxic activities for the twenty target compounds against four cell lines were found to shown moderate activity compared to AAZ, which shows poor cytotoxicity (IC_50_ > 100 μM) with IC_50_ values in the 0.05–125.40 μM range, but the cytotoxic activities were less than the positive control DOX.

**Figure 1:**
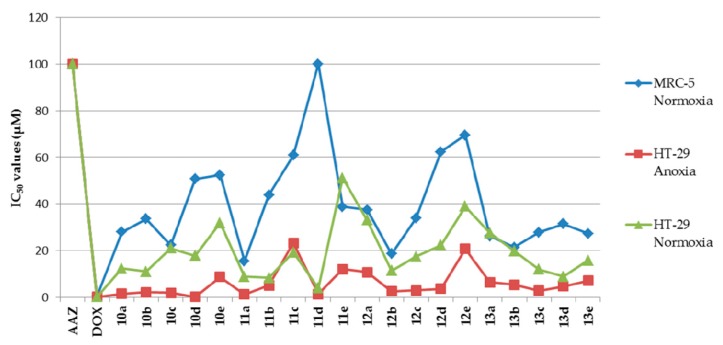
The line chart of the IC_50_ values of tested compounds against MRC-5 and HT-29.

**Figure 2:**
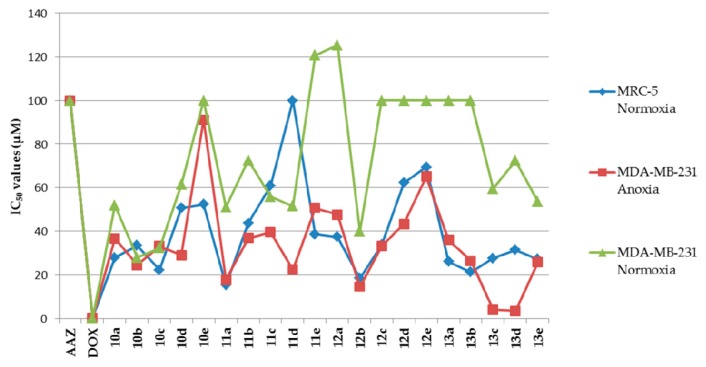
The line chart of the IC_50_ values of tested compounds against MRC-5 and MDA-MB-231.

**Figure 3:**
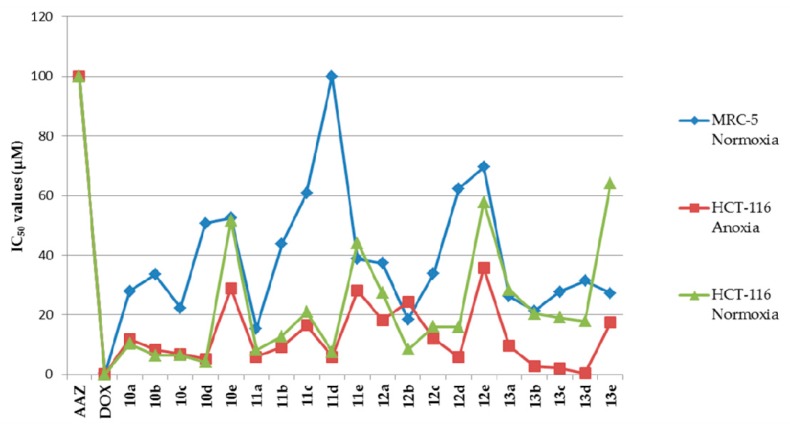
The line chart of the IC_50_ values of tested compounds against MRC-5 and HCT-116.

The line chart that depicted the IC_50_ values was shown in Figure 1, Figure 2 and Figure 3. In order to gain a clearer understanding of the activity data, we set the data “>100” in Table 1 as 100, for it would not affect the result. Then we set out to evaluate the structure-activity relationship (SAR) against three cancer cell lines (HCT-116, MDA-MB-231, and HT-29).

First, we found that for the four series of compounds: (i) in the coumarin ring, the 3-chloro, 4-methyl substituent exhibited better activity; (ii) on the contrary, the 3-ethoxycarbonyl group in the 3-position of coumarin showed poorer activity than others; and (iii) the 4-methyl or 4-trifluoromethyl substituent showed no significant differences in the activity.

Then we analyzed the activity data against three cancer cell lines under normoxic or anoxic condition separately compared with MRC-5 (normal cell line) under normoxic conditions (Figure 1, Figure 2 and Figure 3). In general, the target compounds exhibited better activity against HT-29 cell line under anoxic conditions. The cytotoxicity of these compounds against HT-29 and MDA-MB-231 (CA IX negative [25]) had a general promotion (Figure 1 and Figure 2) under anoxic condition, but the cytotoxicity to MDA-MB-231 exhibited a comparable activity to MRC-5 which mean poor selectivity to normal cells; while the activity for HT-29 under anoxic conditions exhibited better selectivity and appeared less toxic to normal cell MRC-5. For the HCT-116 cell line, which does not express CA IX in response to anoxia [24], there was no significant change in cytotoxicity (Figure 3).

**Figure 4:**
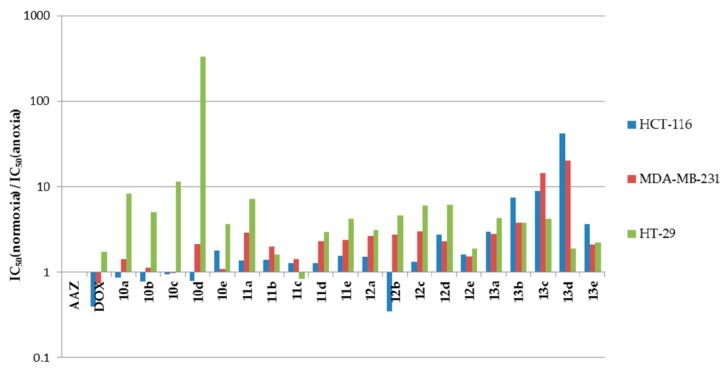
The cytotoxic activities of anoxia condition compared with normoxia codition.

Moreover, the cytotoxic activities of most compounds under anoxic condition displayed one- to 10-fold greater activity than under normoxic condition (Figure 4). For HT-29 cell line which over expresses high amounts of CA IX, the first series **10a**–**e** exhibited better selectivity compared to other two cell lines.

Above all, these results indicated that our design of CA IX inhibitors does correspond with its action mode, and deserved further investigation.

## 3. Materials and Methods

### 3.1. Chemistry

Melting points were recorded on an X-4 microscope melting point apparatus (Beijing Tech Instrument Co., Ltd., Beijing, China) without calibration. The ^1^H-NMR and ^13^C-NMR spectra were measured by a Bruker AV-400 spectrometer (Bruker Bioscience, Billerica, MA, USA), with tetramethylsilane as an internal standard. High-resolution mass spectra (HRMS) were measured with an Agilent Accurate-Mass Q-TOF 6530 (Agilent, Santa Clara, CA, USA) in ESI mode. Reaction progress was monitored by TLC on silica gel precoated GF254 plates (Qingdao Haiyang Chemical Co. Ltd., Qingdao, Shandong, China). Preparative flash column chromatography was performed on the 200–300 mesh silica gel (Qingdao Haiyang Chemical Co. Ltd., Qingdao, Shandong, China). Unless otherwise noted, all solvents and reagents were commercially available and used without further purification.

#### 3.1.1. General Procedure for the Synthesis of Compounds **1a**–**d**

A solution of 1,3-dihydroxybenzene (11.0 g, 100 mmol) in substituted ethyl acetoacetate (100 mmol) was added dropwise to stirring H_2_SO_4_ kept at 0 °C. After completion of the addition, the reaction mixture was kept stirring for 12 h at room temperature and then poured onto ice-water. The crude products were filtrated and recrystallized from ethanol [18].

*7-Hydroxy-4-methyl-2H-chromen-2-one* (**1a**): White solid; yield 73%. ESI-HRMS [M + H]^+^: (*m*/*z*) Calcd. for C_10_H_9_O_3_: 177.0546. Found: 177.0556.

*7-Hydroxy-4-(trifluoromethyl)-2H-chromen-2-one* (**1b**): White solid; yield 52%. ESI-HRMS [M − H]^−^: (*m*/*z*) Calcd. for C_10_H_6_F_3_O_3_: 231.0275. Found: 231.0162.

*7-Hydroxy-3,4-dimethyl-2H-chromen-2-one* (**1c**) White solid; yield 76%. ESI-HRMS [M + H]^+^: (*m*/*z*) Calcd. for C_11_H_11_O_3_: 191.0703. Found: 191.0712.

*3-Chloro-7-hydroxy-4-methyl-2H-chromen-2-one* (**1d**): White solid; yield 74%. ESI-HRMS [M + H]^+^: (*m*/*z*) Calcd. for C_10_H_8_ClO_3_: 211.0156. Found: 211.0166.

#### 3.1.2. The Synthesis of Compound **1e**

2, 4-dihydroxybenzaldehyde (6.9 g, 0.05 mol), diethyl malonate (9.6 g, 0.06 mol) and piperidine (0.2 g, 2.5 mmol) were refluxing in 150 mL ethanol for 10 h. After cooled to room temperature, the crude products were filtrated and recrystallized from methanol [19].

*Ethyl 7-hydroxy-2-oxo-2H-chromene-3-carboxylate* (**1e**): Light yellow solid; yield 81%. ESI-HRMS [M + Na]^+^: (*m*/*z*) Calcd. for C_12_H_10_O_5_Na: 257.0420. Found: 257.0434. 

#### 3.1.3. General procedure for the synthesis of compounds **2a**–**e**, **3a**–**e**

The compounds **2a**–**e** and **3a**–**e** were synthesized according to the method from literature [26,27,28]. To a solution of compound **1a**–**e** (10.0 mmol) in acetone (30 mL), 1,2–dibromoethane or 1,3-dibromopropane (30.0 mmol) and potassium carbonate (12.3 mmol) were added. The reaction mixture was stirred at 56 °C for 10 h, then poured into water, and extracted with ethyl acetate (30 mL × 3). The organic layer was dried over anhydrous Na_2_SO_4_ and concentrated under reduced pressure.

*7-(2-Bromoethoxy)-4-methyl-2H-chromen-2-one* (**2a**): White solid; yield 62%. ESI-HRMS [M + H]^+^: (*m*/*z*) Calcd. for C_12_H_12_BrO_3_: 282.9964. Found: 282.9981.

*7-(2-Bromoethoxy)-4-(trifluoromethyl)-2H-chromen-2-one* (**2b**): White solid; yield 53%. ESI-HRMS [M + H]^+^: (*m*/*z*) Calcd. for C_12_H_9_BrF_3_O_3_: 336.9682. Found: 336.9712.

*7-(2-Bromoethoxy)-3,4-dimethyl-2H-chromen-2-one* (**2c**): White solid; yield 67%. ESI-HRMS [M + H]^+^: (*m*/*z*) Calcd. for C_13_H_14_BrO_3_: 297.0121. Found: 297.0129.

*7-(2-Bromoethoxy)-3-chloro-4-methyl-2H-chromen-2-one* (**2d**): White solid; yield 70%. ESI-HRMS [M + H]^+^: (*m*/*z*) Calcd. for C_12_H_11_BrClO_3_: 316.9575. Found: 316.9589.

*Ethyl 7-(2-bromoethoxy)-2-oxo-2H-chromene-3-carboxylate* (**2e**): Yellow solid; yield 65%. ESI-HRMS [M + H]^+^: (*m*/*z*) Calcd. for C_14_H_14_BrO_5_: 341.0019. Found: 341.0036.

*7-(3-Bromopropoxy)-4-methyl-2H-chromen-2-one* (**3a**): White solid; yield 62%. ESI-HRMS [M + H]^+^: (*m*/*z*) Calcd. for C_13_H_14_BrO_3_: 297.0121. Found: 297.0132.

*7-(3-Bromopropoxy)-4-(trifluoromethyl)-2H-chromen-2-one* (**3b**): White solid; yield 62%. ESI-HRMS [M + H]^+^: (*m*/*z*) Calcd. for C_13_H_11_BrF_3_O_3_: 350.9838. Found: 350.9851.

*7-(3-Bromopropoxy)-3,4-dimethyl-2H-chromen-2-one* (**3c**): White solid; yield 62%. ESI-HRMS [M + H]^+^: (*m*/*z*) Calcd. for C_14_H_16_BrO_3_: 311.0277. Found: 311.0284.

*7-(3-Bromopropoxy)-3-chloro-4-methyl-2H-chromen-2-one* (**3d**): White solid; yield 62%. ESI-HRMS [M + H]^+^: (*m*/*z*) Calcd. for C_13_H_13_BrClO_3_: 330.9731. Found: 330.9748.

*Ethyl 7-(3-bromopropoxy)-2-oxo-2H-chromene-3-carboxylate* (**3e**): Yellow solid; yield 62%. ESI-HRMS [M + H]^+^: (*m*/*z*) Calcd. for C_15_H_16_BrO_5_: 355.0176. Found: 355.0183.

#### 3.1.4. General Procedure for the Synthesis of Compounds **4a**–**e**

To a solution of compound **1a** (10.0 mmol) in DMF (20 mL), propargyl bromide (1.30 g, 10.9 mmol) and potassium carbonate (1.70 g, 12.3 mmol) were added. The reaction mixture was stirred at 60 °C for 5 h, then was diluted with ethyl acetate (80 mL) and washed with water (100 mL × 2). The organic layer was dried over anhydrous Na_2_SO_4_ and concentrated under reduced pressure. The crude products **4a** were used without purification.

The compounds **4b**–**e** were synthesized by the same operation procedure of compound **4a**.

*4-Methyl-7-(prop-2-yn-1-yloxy)-2H-chromen-2-one* (**4a**): White solid; yield 91%; m.p. 129–132 °C. ESI-HRMS [M + H]^+^: (*m*/*z*) Calcd. for C_13_H_10_O_3_: 215.0703. Found: 215.0715.

*7-(Prop-2-yn-1-yloxy)-4-(trifluoromethyl)-2H-chromen-2-one* (**4b**): White solid; yield 78%; m.p. 82–84 °C. ESI-HRMS [M + H]^+^: (*m*/*z*) Calcd. for C_13_H_8_F_3_O_3_: 269.0420. Found: 269.0437.

*3,4-Dimethyl-7-(prop-2-yn-1-yloxy)-2H-chromen-2-one* (**4c**): White solid; yield 85%; m.p. 118–122 °C. ESI-HRMS [M + H]^+^: (*m*/*z*) Calcd. for C_14_H_13_O_3_: 229.0859. Found: 229.0875.

*3-Chloro-4-methyl-7-(prop-2-yn-1-yloxy)-2H-chromen-2-one* (**4d**):White solid; yield 82%; m.p. 131–134 °C. ESI-HRMS [M + H]^+^: (*m*/*z*) Calcd. for C_13_H_10_ClO_3_: 249.0313. Found: 249.0323.

*Ethyl 2-oxo-7-(prop-2-yn-1-yloxy)-2H-chromene-3-carboxylate* (**4e**): White solid; yield 79%; m.p. 122–124 °C. ESI-HRMS [M + H]^+^: (*m*/*z*) Calcd. for C_15_H_13_O_5_: 273.0757. Found: 273.0764.

#### 3.1.5. General Procedure for the Synthesis of Compounds **7a**, **7b**, **9a**

To a solution of DHA (10.0 mmol) and 2-bromoethanol (10.0 mmol) in CH_2_Cl_2_ (30 mL), boron fluoride ethyl ether (0.5 mL) was added dropwise at 0 °C. The reaction mixture was stirred at 0 °C for 8 h, then washed with saturated NaHCO_3_ solution (20 mL × 3). The organic layer was dried over anhydrous Na_2_SO_4_ and concentrated under reduced pressure. The crude products were purified by silica gel column chromatography (PET/EtOAc = 10:1, v/v) to get the target compound **7a**.

The compounds **7b** and **9a** were synthesized by the same operation procedure of compound **7a**.

*1-Bromo-2-(10β-dihydroartemisinoxy)ethane* (**7a**): White solid; yield 87%; m.p. 72–74 °C. ^1^H-NMR (400 MHz, CDCl_3_) δ (ppm): 0.94 (d, *J* = 7.2 Hz, 3H), 0.96 (d, *J* = 6.4 Hz), 1.21–1.40 (m, 3H), 1.44 (s, 3H), 1.45–1.56 (m, 2H), 1.63–1.68 (m, 1H), 1.73–1.79 (m, 1H), 1.84–1.96 (m, 2H), 2.01–2.07 (m, 1H), 2.33–2.41 (m, 1H), 2.61–2.69 (m, 1H), 3.52 (t, *J* = 5.2 Hz, 2H), 3.77–3.82 (m, 1H), 4.09–4.15 (m, 1H), 4.85 (d, *J* = 3.2 Hz, 1H), 5.49 (s, 1H). ESI-HRMS [M + Na]^+^: (*m*/*z*) Calcd. for C_17_H_27_BrO_5_Na: 413.0934. Found: 413.0942.

*1-Bromo-3-(10β-dihydroartemisinoxy)propane (**7b***): White solid; yield 89%; m.p. 68–71 °C. ^1^H-NMR (400 MHz, CDCl_3_) δ (ppm): 0.92 (d, *J* = 7.6 Hz, 3H), 0.96 (d, *J* = 6.0 Hz, 3H), 1.19–1.28 (m, 1H), 1.29–1.39 (m, 1H), 1.44 (s, 3H), 1.45–1.56 (m, 2H), 1.60–1.69 (m, 1H), 1.72–1.78 (m, 2H), 1.85–1.92 (m, 1H), 2.01–2.14 (m, 3H), 2.37 (dt, *J* = 4.0 Hz, *J* = 13.6 Hz, 1H), 2.60–2.68 (m, 1H), 3.47–3.52 (m, 3H), 3.98–4.03 (m, 1H), 4.81 (d, *J* = 3.6 Hz, 1H), 5.43 (s, 1H). ESI-HRMS [M + Na]^+^: (*m*/*z*) Calcd. for C_18_H_29_BrO_5_Na: 427.1091. Found: 427.1099.

*3-(10β-Dihydroartemisinoxy)prop-1-yne (**9a**)*: White solid; yield 87%; m.p. 105–108 °C. ^1^H-NMR (400 MHz, CDCl_3_) δ (ppm): 0.93 (d, *J* = 7.6 Hz, 3H), 0.95 (d, *J* = 6.8 Hz, 3H), 1.20–1.40 (m, 3H), 1.44 (s, 3H), 1.47–1.55 (m, 2H), 1.62–1.66 (m, 1H), 1.74–1.79 (m, 2H), 1.85–1.92 (m, 1H), 2.01–2.07 (m, 1H), 2.33–2.41 (m, 2H), 2.64–2.71 (m, 1H), 4.31 (d, *J* = 1.2 Hz, 2H), 4.98 (d, *J* = 3.2 Hz), 5.42 (s, 1H). ESI-HRMS [M + Na]^+^: (*m*/*z*) Calcd. for C_18_H_26_O_5_Na: 345.1672. Found: 345.1683.

#### 3.1.6. General Procedure for the Synthesis of Compounds **5a**–**e**, **6a**–**e**, **8a**, **8b**

These compounds were synthesized by the same operation in literature [20]. To a solution of compound **2a** (10 mmol) in DMF (20 mL), sodium azide (30 mmol) was added, the reaction mixture was stirred at 70 °C for 4 h. the reaction mixture was poured into water and extracted with ethyl acetate (40 mL × 3). The organic layer was dried over anhydrous Na_2_SO_4_, concentrated under reduced pressure, and the crude products were used in next step immediately without further purification.

#### 3.1.7. General Procedure for the Synthesis of Compounds **10a**–**e**, **11a**–**e**, **12a**–**e**, **13a**–**e**

To a solution of compound **8a** (10 mmol) and **4a** (10 mmol) in CH_2_Cl_2_ (20 mL), a mixture of CuSO_4_·5H_2_O (12.5 mg) and sodium ascorbate (30 mg) in water (10 mL) was added, and the reaction mixture was stirred at room temperature for 8 h. The reaction mixture was filtered and the organic layer was washed with water, then dried over anhydrous Na_2_SO_4_ and concentrated under reduced pressure. The crude product was purified by silica gel column chromatography (PET/EtOAc = 2:1, *v/v*) to get the target compound **10a**.

The compounds **10b**–**e**, **11a**–**e**, **12a**–**e**, **13a**–**e** were synthesized by the same operation procedure of compound **10a** (**5a**–**e**, **6a**–**e** with **9a**; **8a**, **8b** with **4a**–**e**).

*7-(2-(4-(10β-Dihydroartemisininoxy)methyl)-1H-1,2,3-triazol-1-yl)ethoxy-4-methyl-2H-chromen-2-one* (**10a**): White solid; yield 31%; m.p. 83–85 °C. ^1^H-NMR (400 MHz, CDCl_3_) δ (ppm): 0.87 (d, *J* = 7.6 Hz, 3H), 0.92 (d, *J* = 6.4 Hz, 3H), 1.19–1.28 (m, 3H), 1.44 (s, 3H), 1.45–1.50 (m, 1H), 1.55–1.59 (m, 1H), 1.70–1.75 (m, 1H), 1.80–1.90 (m, 1H), 1.99–2.04 (m, 1H), 2.33–2.37 (m, 1H), 2.40 (s, 3H), 2.60–2.68 (m, 1H), 4.43–4.46 (m, 2H), 4.76–4.71 (d, *J* = 12.4 Hz, 2H), 4.80–4.83 (m, 2H), 4.91 (d, *J* = 3.6 Hz, 1H), 4.95 (d, *J* = 12.4 Hz, 1H), 5.42 (s, 1H), 6.16 (s, 1H), 6.79 (d, *J* = 2.4 Hz, 1H), 6.80–6.83 (dd, *J* = 2.4 Hz, *J* = 8.8 Hz, 1H), 7.50 (d, *J* = 8.8 Hz, 1H), 7.69 (s, 1H). ^13^C-NMR (100 MHz, CDCl_3_) δ (ppm): 160.90, 160.65, 155.12, 152.29, 145.40, 125.85, 123.49, 114.39, 112.56, 112.07, 104.13, 101.77, 101.58, 87.98, 81.06, 66.80, 61.61, 52.51, 49.41, 44.35, 37.36, 36.40, 34.56, 30.78, 26.14, 24.64, 24.42, 20.31, 18.65, 12.97. ESI-HRMS [M + Na]^+^: (*m*/*z*) Calcd. for C_30_H_37_N_3_O_8_Na: 590.2473. Found: 590.2462. 

*7-(2-(4-(10β-Dihydroartemisininoxy)methyl)-1H-1,2,3-triazol-1-yl)ethoxy-4-trifluoromethyl-2H-chromen-2-one* (**10b**): White solid; yield 29%; m.p. 69–71 °C. ^1^H-NMR (400 MHz, CDCl_3_) δ (ppm): 0.88 (d, *J* = 7.2 Hz, 3H), 0.92 (d, *J* = 6.0 Hz, 3H), 1.19–1.30 (m, 3H), 1.44 (s, 3H), 1.55–1.59 (m, 1H), 1.62–1.80 (m, 3H), 1.84–2.05 (m, 3H), 2.33–2.41 (m, 1H), 2.61–2.68 (m, 1H), 4.48 (t, *J* = 4.8 Hz, 2H), 4.69 (d, *J* = 12.4 Hz, 1H), 4.84 (m, 2H), 4.91 (d, *J* = 3.6 Hz, 1H), 4.95 (d, *J* = 12.4 Hz, 1H), 5.42 (s, 1H), 6.66 (s, 1H), 6.86 (d, *J* = 2.8 Hz, 1H), 6.89 (dd, *J* = 2.4 Hz, *J* = 9.2 Hz, 1H), 7.63 (d, *J* = 8.8 Hz, 1H), 7.69 (s, H). ^13^C-NMR (100 MHz, CDCl_3_) δ (ppm): 161.42, 158.89, 156.07, 145.40, 130.83, 128.74, 126.57, 123.37, 113.10, 112.97, 112.93, 112.90, 112.86, 107.73, 104.06, 102.12, 101.52, 87.90, 80.97, 66.88, 65.47, 61.53, 52.40, 49.16, 44.24, 36.29, 34.46, 30.68, 26.05, 24.55, 24.33, 20.21, 12.88. ESI-HRMS [M + Na]^+^: (*m*/*z*) Calcd. for C_30_H_34_F_3_N_3_O_8_Na: 644.2190. Found: 644.2183.

*7-(2-(4-(10β-Dihydroartemisininoxy)methyl)-1H-1,2,3-triazol-1-yl)ethoxy-3,4-dimethyl-2H-chromen-2-one* (**10c**): White solid; yield 37%; m.p. 82–84 °C. ^1^H-NMR (400 MHz, CDCl_3_) δ (ppm): 0.87 (d, *J* = 7.2 Hz, 3H), 0.92 (d, *J* = 6.0 Hz, 3H), 1.20–1.31 (m, 3H), 1.44 (s, 3H), 1.47–1.50 (m, 1H), 1.55–1.59 (m, 1H), 1.71–1.78 (m, 2H), 1.83–1.89 (m, 1H), 2.00–2.13 (m, 2H), 2.19 (s, 3H), 2.32–2.40 (m, 1H), 2.37 (s, 3H), 2.6–2.68 (m, 1H), 4.43 (t, *J* = 4.8 Hz, 2H), 4.68 (d, *J* = 12.8 Hz, 1H), 4.80–4.83 (m, 2H), 4.91 (d, *J* = 3.6 Hz, 1H), 4.95 (d, *J* = 12.8 Hz, 1H), 5.42 (s, 1H), 6.78 (s, 1H), 6.80 (dd, *J* = 2.4 Hz, *J* = 8.8 Hz, 1H), 7.50 (d, *J* = 8.8 Hz, 1H), 7.70 (s, 1H). ^13^C-NMR (100 MHz, CDCl_3_) δ (ppm): 162.13, 159.52, 153.41, 145.96, 145.41, 125.56, 123.49, 119.67, 115.01, 111.87, 104.14, 101.60, 101.43, 87.99, 81.09, 66.71, 61.65, 52.51, 49.45, 44.34, 37.37, 36.40, 34.56, 30.79, 26.18, 24.64, 24.43, 20.34, 15.18, 13.23, 13.00. ESI-HRMS [M + Na]^+^: (*m*/*z*) Calcd. for C_31_H_39_N_3_O_8_Na: 604.2629. Found: 604.2636.

*3-Chloro-7-(2-(4-(10β-dihydroartemisininoxy)methyl)-1H-1,2,3-triazol-1-yl)ethoxy-4-methyl-2H-chromen-2-one* (**10d**): White solid; yield 39%; m.p. 75–77 °C. ^1^H-NMR (400 MHz, CDCl_3_) δ (ppm): 0.87 (d, *J* = 7.2 Hz, 3H), 0.92 (d, *J* = 6.4 Hz, 3H), 1.21–1.28 (m, 3H), 1.44 (s, 3H), 1.47–1.50 (m, 1H), 1.55–1.59 (m, 1H), 1.71–1.75 (m, 2H), 1.84–1.89 (m, 1H), 2.00–2.05 (m, 2H), 2.32–2.40 (m, 1H), 2.54 (s, 3H), 2.61–2.65 (m, 1H), 4.46 (m, 2H), 4.68–4.70 (d, *J* = 12.4 Hz, 2H), 4.81–4.84 (m, 2H), 4.90–4.91 (d, *J* = 3.6 Hz, 1H), 4.93–4.96 (d, *J* = 12.4 Hz, 1H), 5.42 (s, 1H), 6.80 (d, *J* = 2.4 Hz, 1H), 6.87 (dd, *J* = 2.4 Hz, *J* = 8.8 Hz, 1H), 7.53 (d, *J* = 8.8 Hz, 1H), 7.70 (s, 1H). ^13^C-NMR (100 MHz, CDCl_3_) δ (ppm): 160.55, 157.08, 152.92, 147.70, 145.39, 126.18, 123.51, 118.42, 114.07, 112.76, 104.14, 101.67, 101.55, 87.99, 81.06, 66.88, 61.57, 52.50, 49.36, 44.34, 37.37, 36.39, 34.56, 30.77, 26.16, 24.65, 24.43, 20.33, 16.19, 12.98. ESI-HRMS [M + Na]^+^: (*m*/*z*) Calcd. for C_30_H_36_ClN_3_O_8_Na: 624.2083. Found: 624.2088.

*Ethyl 2-oxo-7-(2-(4-(10β-dihydroartemisininoxy)methyl)-1H-1,2,3-triazol-1-yl)ethoxy-2H-chromene-3-carboxylate* (**10e**): White solid; yield 23%; m.p. 153–155 °C. ^1^H-NMR (400 MHz, CDCl_3_) δ (ppm): 0.87 (d, *J* = 7.2 Hz, 3H), 0.92 (d, *J* = 6.4 Hz, 3H), 1.19–1.30 (m, 3H), 1.40 (t, *J* = 6.8 Hz, 3H), 1.44 (s, 3H), 1.46–1.60 (m, 3H), 1.70–1.78 (m, 2H), 1.83–1.90 (m, 1H), 2.00–2.06 (m, 1H), 2.33–2.41 (m, 1H), 2.61–2.68 (m, 1H), 4.40 (q, *J* = 7.2 Hz, 2H), 4.48 (t, *J* = 4.8 Hz, 2H), 4.69 (d, *J* = 12.8 Hz, 1H), 4.84 (m, 2H), 4.91 (d, *J* = 3.2 Hz, 1H), 4.95 (d, *J* = 12.4 Hz, 1H), 5.42 (s, 1H), 6.80 (d, *J* = 2.4 Hz, 1H), 6.86 (dd, *J* = 2.4 Hz, *J* = 8.8 Hz, 1H), 7.52 (d, *J* = 8.8 Hz, 1H), 7.69 (s, 1H), 8.50 (s, 1H). ^13^C-NMR (100 MHz, CDCl_3_) δ (ppm): 163.21, 162.98, 157.26, 156.79, 148.62, 145.44, 130.98, 123.49, 114.93, 113.35, 112.33, 104.15, 101.58, 101.21, 87.99, 81.07, 67.01, 61.82, 61.58, 52.49, 49.25, 44.32, 37.38, 36.38, 34.55, 30.77, 26.17, 24.65, 24.43, 20.34, 14.27, 12.99. ESI-HRMS [M + Na]^+^: (*m*/*z*) Calcd. for C_32_H_39_N_3_O_10_Na: 648.2528. Found: 648.2525. 

*7-(3-(4-(10β-Dihydroartemisininoxy)methyl)-1H-1,2,3-triazol-1-yl)propoxy-4-methyl-2H-chromen-2-one* (**11a**): White solid; yield 32%; m.p. 69–70 °C. ^1^H-NMR (400 MHz, CDCl_3_) δ (ppm): 0.84 (d, *J* = 7.6 Hz, 3H), 0.92 (d, *J* = 6.0 Hz, 3H), 1.21–1.28 (m, 3H), 1.44 (s, 3H), 1.46–1.50 (m, 1H), 1.55–1.59 (m, 1H), 1.65–1.76 (m, 3H), 1.84–1.89 (m, 1H), 1.99–2.05 (m, 2H), 2.41 (s, 3H), 2.46 (m, 2H), 2.61–2.64 (m, 1H), 4.04 (t, *J* = 5.6 Hz, 2H), 4.60–4.63 (m, 2H), 4.67 (d, *J* = 12.8 Hz, 1H), 4.89 (d, *J* = 3.2 Hz, 1H), 4.93 (d, *J* = 12.4 Hz, 1H), 5.39 (s, 1H), 6.16 (s, 1H), 6.79 (d, *J* = 2.0 Hz, 1H), 6.84 (dd, *J* = 3.2 Hz, *J* = 8.8 Hz, 1H), 7.50 (d, *J* = 3.2 Hz, 1H), 7.51 (d, *J* = 5.2 Hz, 1H). ^13^C-NMR (100 MHz, CDCl_3_) δ (ppm): 161.35, 161.11, 155.21, 152.43, 145.23, 125.75, 122.83, 114.02, 112.29, 112.11, 104.13, 101.71, 101.66, 87.96, 81.08, 64.69, 61.72, 52.49, 46.87, 44.34, 37.38, 36.40, 34.55, 30.79, 29.70, 26.16, 24.67, 24.42, 20.32, 18.69, 12.96. ESI-HRMS [M + Na]^+^: (*m*/*z*) Calcd. for C_31_H_39_N_3_O_8_Na: 604.2629. Found: 604.2618. 

*7-(3-(4-(10β-Dihydroartemisininoxy)methyl)-1H-1,2,3-triazol-1-yl)propoxy-4-trifluoromethyl-2H-chromen-2-one* (**11b**): White solid; yield 19%; m.p. 67–69 °C. ^1^H-NMR (400 MHz, CDCl_3_) δ (ppm): 0.85 (d, *J* = 7.2 Hz, 3H), 0.92 (d, *J* = 6.0 Hz, 3H), 1.19–1.29 (m, 3H), 1.44 (s, 3H), 1.46–1.50 (m, 2H), 1.55–1.59 (m, 1H), 1.66–1.76 (m, 2H), 1.84–1.89 (m, 1H), 1.99–2.05 (m, 1H), 2.32–2.40 (m, 1H), 2.48 (m, 2H), 2.59–2.67 (m, 1H), 4.07 (t, *J* = 6.0 Hz, 2H), 4.60–4.64 (m, 2H), 4.68 (d, *J* = 12.4 Hz, 1H), 4.90 (d, *J* = 3.2 Hz, 1H), 4.93 (d, *J* = 12.4 Hz, 1H), 5.39 (s, 1H), 6.64 (s, 1H), 6.85 (d, *J* = 2.8 Hz, 1H), 6.91 (dd, *J* = 2.4 Hz, *J* = 9.2 Hz, 1H), 7.52 (s, 1H), 7.64 (dd, *J* = 1.6 Hz, *J* = 8.8 Hz, 1H). ^13^C-NMR (100 MHz, CDCl_3_) δ (ppm): 162.23, 159.19, 156.25, 145.29, 126.57, 122.807, 120.19, 113.27, 112.66, 112.60, 107.44, 104.14, 102.10, 101.71, 87.96, 81.07, 64.95, 61.70, 52.48, 46.76, 44.33, 37.38, 36.39, 34.54, 30.78, 29.60, 26.15, 24.67, 24.41, 20.31, 12.95. ESI-HRMS [M + Na]^+^: (*m*/*z*) Calcd. for C_31_H_36_F_3_N_3_O_8_Na: 658.2347. Found: 658.2350. 

*7-(3-(4-(10β-Dihydroartemisininoxy)methyl)-1H-1,2,3-triazol-1-yl)propoxy-3,4-dimethyl-2H-chromen-2-one* (**11c**): White solid; yield 35%; m.p. 83–85 °C. ^1^H-NMR (400 MHz, CDCl_3_) δ (ppm): 0.85 (d, *J* = 5.2 Hz, 3H), 0.87 (d, *J* = 6.0 Hz, 3H), 0.90–0.99 (m, 1H), 1.15–1.27 (m, 2H), 1.30–1.31 (m, 1H), 1.55 (s, 3H), 1.58–1.72 (m, 3H), 1.74–1.79 (m, 1H), 1.82–1.89 (m, 1H), 2.19 (s, 3H), 2.38 (s, 3H), 2.40–2.48 (m, 3H), 3.57 (m, 1H), 4.00–4.03 (m, 2H), 4.59–4.65 (m, 3H), 4.87 (d, *J* = 4.4 Hz, 1H), 4.90 (d, *J* = 8.8 Hz, 1H), 5.26 (s, 1H), 6.77 (d, *J* = 2.4 Hz, 1H), 6.83 (dd, *J* = 2.8 Hz, *J* = 8.8 Hz, 1H), 7.49 (s, 1H), 7.51 (d, *J* = 9.2 Hz, 1H). ^13^C-NMR (100 MHz, CDCl_3_) δ (ppm): 162.30, 160.24, 153.48, 146.11, 145.32, 125.44, 122.75, 119.31, 114.61, 111.96, 107.99, 101.32, 99.46, 93.80, 84.08, 77.26, 69.56, 64.53, 61.81, 46.90, 42.41, 40.63, 34.82, 34.70, 30.32, 29.72, 24.98, 20.99, 18.82, 15.10, 13.19, 12.26. ESI-HRMS [M + Na]^+^: (*m*/*z*) Calcd. for C_32_H_41_N_3_O_8_Na: 618.2786. Found: 618.2784.

*3-Chloro-7-(3-(4-(10β-dihydroartemisininoxy)methyl)-1H-1,2,3-triazol-1-yl)propoxy-4-methyl-2H-chromen-2-one* (**11d**): White solid; yield 34%; m.p. 150–152 °C. ^1^H-NMR (400 MHz, CDCl_3_) δ (ppm): 0.85 (d, *J* = 7.2 Hz, 3H), 0.92 (d, *J* = 6.0 Hz, 3H), 1.19–1.28 (m, 3H), 1.44 (s, 3H), 1.45–1.49 (m, 1H), 1.55–1.59 (m, 1H), 1.65–1.81 (m, 3H), 1.83–1.90 (m, 1H), 1.99–2.05 (m, 1H), 2.32–2.40 (m, 1H), 2.47 (m, 2H), 2.56 (s, 3H), 2.59–2.66 (m, 1H), 4.05 (m, 2H), 4.61 (m, 2H), 4.67 (d, *J* = 12.8 Hz, 1H), 4.89 (d, *J* = 3.2 Hz, 1H), 4.92 (d, *J* = 12.8 Hz, 1H), 5.39 (s, 1H), 6.80 (d, *J* = 2.4 Hz, 1H), 6.88 (dd, *J* = 2.4 Hz, *J* = 8.8 Hz, 1H), 7.52 (s, 1H), 7.54 (d, *J* = 8.8 Hz, 1H). ^13^C-NMR (100 MHz, CDCl_3_) δ (ppm): 161.26, 157.26, 153.01, 147.84, 145.22, 126.09, 122.84, 118.10, 113.68, 112.81, 104.13, 101.67, 101.56, 87.96, 81.07, 64.84, 61.67, 52.48, 46.86, 44.33, 37.38, 36.39, 34.55, 30.78, 29.68, 26.16, 24.67, 24.42, 20.33, 16.21, 12.97. ESI-HRMS [M + Na]^+^: (*m*/*z*) Calcd. for C_31_H_38_ClN_3_O_8_Na: 638.2240. Found: 638.2243. 

*Ethyl 2-oxo-7-(3-(4-(10β-dihydroartemisininoxy)methyl)-1H-1,2,3-triazol-1-yl)propoxy-2H-chromene-3-carboxylate* (**11e**): White solid; yield 29%; m.p. 124–126 °C. ^1^H-NMR (400 MHz, CDCl_3_) δ (ppm): 0.85 (d, *J* = 7.2 Hz, 3H), 0.92 (d, *J* = 6.0 Hz, 3H), 1.20–1.32 (m, 3H), 1.41 (t, *J* = 7.2 Hz, 3H), 1.44 (s, 3H), 1.46–1.50 (m, 1H), 1.55–1.59 (m, 1H), 1.64–1.81 (m, 3H), 1.83–1.90 (m, 1H), 1.99–2.05 (m, 1H), 2.32–2.40 (m, 1H), 2.48 (m, 2H), 2.59–2.67 (m, 1H), 4.08 (t, *J* = 6.0 Hz, 2H), 4.41 (q, *J* = 7.2 Hz, 2H), 4.61 (td, *J* = 2.0 Hz, *J* = 6.8 Hz, 2H), 4.68 (d, *J* = 12.4 Hz, 1H), 4.90 (d, *J* = 3.2 Hz, 1H), 4.93 (d, *J* = 12.8 Hz, 1H), 5.39 (s, 1H), 6.79 (d, *J* = 2.0 Hz, 1H), 6.87 (dd, *J* = 2.4 Hz, *J* = 8.8 Hz, 1H), 7.52 (d, *J* = 6.4 Hz, 1H), 7.53 (d, *J* = 2.4 Hz, 1H), 8.50 (s, 1H). ^13^C-NMR (100 MHz, CDCl_3_) δ (ppm): 163.78, 163.31, 157.37, 156.96, 148.80, 145.26, 130.91, 122.83, 114.48, 113.45, 111.97, 104.13, 101.69, 101.12, 87.95, 81.07, 65.11, 61.76, 61.68, 52.47, 46.82, 44.32, 37.37, 36.381, 34.54, 30.78, 29.62, 26.16, 24.66, 24.41, 20.32, 14.29, 12.97. ESI-HRMS [M + Na]^+^: (*m*/*z*) Calcd. for C_33_H_41_N_3_O_10_Na: 662.2684. Found: 662.2679.

*7-(1-(2-(10β-Dihydroartemisininoxy)ethyl)-1H-1,2,3-triazol-4-yl)methoxy-4-methyl-2H-chromen-2-one* (**12a**): White solid; yield 32%; m.p. 79–81 °C. ^1^H-NMR (400 MHz, CDCl_3_) δ (ppm): 0.78 (d, *J* = 7.2 Hz, 3H), 0.92 (d, *J* = 5.6 Hz, 3H), 1.16–1.30 (m, 3H), 1.36–1.39 (m, 1H), 1.42 (s, 3H), 1.45–1.52 (m, 2H), 1.56–1.60 (m, 1H), 1.64–1.74 (m, 1H), 1.84–1.88 (m, 1H), 1.99–2.05 (m, 1H), 2.31–2.39 (m, 1H), 2.41 (s, 3H), 2.57–2.64 (m, 1H), 3.79–3.84 (m, 1H), 4.27–4.32 (m, 1H), 4.51–4.57 (m, 1H), 4.63–4.69 (m, 1H), 4.76 (d, *J* = 3.2 Hz, 1H), 5.15 (s, 1H), 5.27 (s, 2H), 6.16 (s, 1H), 6.92 (d, *J* = 2.0 Hz, 1H), 6.95 (dd, *J* = 2.4 Hz, *J* = 8.8 Hz, 1H), 7.52 (d, *J* = 8.8 Hz, 1H), 7.72 (s, 1H). ^13^C-NMR (100 MHz, CDCl_3_) δ (ppm): 161.13, 155.15, 152.42, 143.10, 125.73, 123.49, 114.09, 112.33, 112.31, 104.21, 102.18, 102.13, 87.87, 80.80, 77.24, 66.40, 62.33, 52.38, 50.50, 44.04, 37.34, 36.31, 34.45, 30.59, 26.10, 24.61, 24.34, 20.36, 18.69, 12.81. ESI-HRMS [M + Na]^+^: (*m*/*z*) Calcd. for C_30_H_37_N_3_O_8_Na: 590.2473. Found: 590.2471.

*7-(1-(2-(10β-Dihydroartemisininoxy)ethyl)-1H-1,2,3-triazol-4-yl)methoxy-4-trifluoromethyl-2H-chromen-2-one* (**12b**): White solid; yield 24%; m.p. 77–79 °C. ^1^H-NMR (400 MHz, CDCl_3_) δ (ppm): 0.78 (d, *J* = 7.2 Hz, 3H), 0.92 (d, *J* = 5.6 Hz, 3H), 1.16–1.31 (m, 3H), 1.42 (s, 3H), 1.48–1.76 (m, 4H), 1.84–1.88 (m, 1H), 2.00–2.05 (m, 1H), 2.31–2.34 (m, 1H), 2.59–2.62 (m, 1H), 3.80–3.85 (m, 1H), 4.25–4.32 (m, 1H), 4.51–4.70 (m, 2H), 4.76 (d, *J* = 2.7 Hz, 1H), 5.16 (s, 1H), 5.29 (s, 1H), 6.64 (s, 1H), 7.01–7.03 (m, 2H), 7.64 (d, *J* = 9.2 Hz, 1H), 7.73 (s, 1H). ^13^C-NMR (100 MHz, CDCl_3_) δ (ppm): 161.928, 159.095, 156.088, 142.555, 126.430, 124.988, 123.535, 113.398, 112.573, 107.385, 104.107, 102.503, 102.073, 87.770, 80.677, 77.191, 76.979, 76.767, 66.282, 62.356, 52.277, 50.429, 43.935, 37.235, 36.202, 34.358, 30.480, 25.974, 24.517, 24.229, 20.241, 19.083, 12.691. ESI-HRMS [M + Na]^+^: (*m*/*z*) Calcd. for C_30_H_34_F_3_N_3_O_8_Na: 644.2190. Found: 644.2187.

*7-(1-(2-(10β-Dihydroartemisininoxy)ethyl)-1H-1,2,3-triazol-4-yl)methoxy-3,4-dimethyl-2H-chromen-2-one* (**12c**): White solid; yield 36%; m.p. 84–86 °C. ^1^H-NMR (400 MHz, CDCl_3_) δ (ppm): 0.78 (d, *J* = 7.2 Hz, 3H), 0.91 (d, *J* = 4.4 Hz, 3H), 0.97–1.29 (m, 3H), 1.42 (s, 3H), 1.45–1.55 (m, 2H), 1.55–2.05 (m, 5H), 2.18 (s, 3H), 2.30–2.34 (m, 1H), 2.37 (s, 3H), 2.57–2.61 (m, 1H), 3.79–3.84 (m, 1H), 4.27–4.32 (m, 1H), 4.51–4.57 (m, 1H), 4.63–4.69 (m, 1H), 4.76 (d, *J* = 3.2 Hz, 1H), 5.15 (m, 1H), 5.25 (m, 2H), 6.90–6.95 (m, 2H), 7.52 (dd, *J* = 3.6 Hz, *J* = 8.8 Hz, 1H), 7.72 (s, 1H). ^13^C-NMR (100 MHz, CDCl_3_) δ (ppm): 162.26, 160.01, 153.39, 146.12, 143.21, 125.44, 123.52, 119.29, 114.66, 112.10, 104.16, 102.13, 101.76, 87.84, 80.78, 66.38, 62.25, 52.37, 50.47, 44.03, 37.29, 36.30, 34.43, 30.58, 26.07, 24.58, 24.31, 20.34, 15.08, 13.17, 12.80. ESI-HRMS [M + Na]^+^: (*m*/*z*) Calcd. for C_31_H_39_N_3_O_8_Na: 604.2629. Found: 604.2645.

*3-Chloro-7-(1-(2-(10β-dihydroartemisininoxy)ethyl)-1H-1,2,3-triazol-4-yl)methoxy-4-methyl-2H-chromen-2-one* (**12d**): White solid; yield 33%; m.p. 76–78 °C. ^1^H-NMR (400 MHz, CDCl_3_) δ (ppm): 0.78 (d, *J* = 7.2 Hz, 3H), 0.92 (d, *J* = 5.6 Hz, 3H), 1.16–1.32 (m, 3H), 1.32–1.42 (m, 2H), 1.42 (s, 3H), 1.44–1.69 (m, 5H), 1.84–1.88 (m, 1H), 1.99–2.15 (m, 2H), 2.31–2.39 (m, 1H), 2.58–2.62 (m, 1H), 3.79–3.85 (m, 1H), 4.27–4.32 (m, 1H), 4.51–4.58 (m, 1H), 4.63–4.69 (m, 1H), 4.76 (d, *J* = 3.2 Hz, 1H), 5.15 (s, 1H), 5.27 (s, 2H), 6.94 (d, *J* = 2.4 Hz, 1H), 7.00 (dd, *J* = 2.4 Hz, *J* = 8.8 Hz, 1H), 7.55 (d, *J* = 8.8 Hz, 1H), 7.73 (s, 1H). ^13^C-NMR (100 MHz, CDCl_3_) δ (ppm): 161.04, 157.29, 152.97, 147.83, 142.90, 126.07, 123.56, 118.17, 113.78, 113.02, 104.21, 102.18, 102.06, 87.87, 80.79, 66.40, 62.38, 52.38, 50.51, 44.03, 37.33, 36.31, 34.46, 30.59, 26.09, 24.61, 24.34, 20.36, 16.20, 12.82. ESI-HRMS [M + Na]^+^: (*m*/*z*) Calcd. for C_30_H_36_ClN_3_O_8_Na: 624.2083. Found: 624.2111.

*Ethyl 2-oxo-7-(1-(2-(10β-dihydroartemisininoxy)ethyl)-1H-1,2,3-triazol-4-yl)methoxy-2H-chromene-3-carboxylate* (**12e**): White solid; yield 26%; m.p. 71–73 °C. ^1^H-NMR (400 MHz, CDCl_3_) δ (ppm): 0.78 (d, *J* = 7.6 Hz, 3H), 0.92 (d, *J* = 5.6 Hz, 3H), 1.16–1.28 (m, 3H), 1.39–1.42 (m, 8H), 1.47–1.52 (m, 1H), 1.55–1.60 (m, 1H), 1.65–1.69 (m, 1H), 1.84–1.89 (m, 1H), 1.99–2.05 (m, 1H), 2.31–2.39 (m, 1H), 2.57–2.64 (m, 1H), 3.80–3.85 (m, 1H), 4.27–4.32 (m, 1H), 4.40 (q, *J* = 3.2 Hz, 2H), 4.52–4.58 (m, 1H), 4.64–4.70 (m, 1H), 4.76 (d, *J* = 3.6 Hz, 1H), 5.15 (s, 1H), 5.29 (s, 2H), 6.94 (d, *J* = 2.0 Hz, 1H), 6.98 (dd, *J* = 2.4 Hz, *J* = 8.8 Hz, 1H), 7.52 (d, *J* = 8.8 Hz, 1H), 7.74 (s, 1H), 8.51 (s, 1H). ^13^C-NMR (100 MHz, CDCl_3_) δ (ppm): 163.55, 163.36, 157.34, 156.98, 148.79, 142.60, 130.88, 123.64, 114.63, 113.63, 112.08, 104.23, 102.19, 101.62, 87.88, 80.78, 66.40, 62.52, 61.79, 52.37, 50.54, 44.02, 37.35, 36.31, 34.46, 30.58, 26.10, 24.62, 24.34, 20.36, 14.29, 12.82. ESI-HRMS [M + Na]^+^: (*m*/*z*) Calcd. for C_32_H_39_N_3_O_10_Na: 648.2528. Found: 648.2530. 

*7-(1-(3-(10β-Dihydroartemisininoxy)propyl)-1H-1,2,3-triazol-4-yl)methoxy-4-methyl-2H-chromen-2-one* (**13a**): White solid; yield 39%; m.p. 75–77 °C. ^1^H-NMR (400 MHz, CDCl_3_) δ (ppm): 0.93 (d, *J* = 7.2 Hz, 3H), 0.96 (d, *J* = 6.4 Hz, 3H), 1.22–1.33 (m, 3H), 1.43 (s, 3H), 1.46–1.55 (m, 2H), 1.63–1.69 (m, 1H), 1.76–1.78 (m, 1H), 1.86–1.93 (m, 1H), 2.02–2.13 (m, 2H), 2.17–2.25 (m, 2H), 2.33–2.38 (m, 1H), 2.41 (s, 3H), 2.62–2.69 (m, 1H), 3.36–3.42 (m, 1H), 3.87–3.93 (m, 1H), 4.44–4.52 (m, 2H),4.77 (d, *J* = 3.6 Hz, 1H), 5.27 (s, 2H), 5.40 (s, 1H), 6.15 (s, 1H), 6.93 (d, *J* = 2.0 Hz, 1H), 6.96 (dd, *J* = 2.4 Hz, *J* = 8.8 Hz, 1H), 7.52 (d, *J* = 8.8 Hz, 1H), 7.66 (s, 1H). ^13^C-NMR (100 MHz, CDCl_3_) δ (ppm): 161.18, 161.13, 155.12, 152.50, 143.05, 125.74, 123.05, 114.07, 112.39, 112.29, 104.19, 102.19, 102.13, 87.94, 80.97, 64.65, 62.311, 52.50, 47.65, 44.27, 37.46, 36.37, 34.56, 30.81, 30.44, 26.15, 24.66, 24.55, 20.36, 18.70, 13.09. ESI-HRMS [M + Na]^+^: (*m*/*z*) Calcd. for C_31_H_39_N_3_O_8_Na: 604.2629. Found: 604.2674.

*7-(1-(3-(10β-Dihydroartemisininoxy)propyl)-1H-1,2,3-triazol-4-yl)methoxy-4-trifluoromethyl-2H-chromen-2-one* (**13b**): White solid; yield 25%; m.p. 69–71 °C. ^1^H-NMR (400 MHz, CDCl_3_) δ (ppm): 0.94 (d, *J* = 7.2 Hz, 3H), 0.97 (d, *J* = 6.0 Hz, 3H), 1.22–1.39 (m, 3H), 1.43 (s, 3H), 1.46–1.55 (m, 2H), 1.64–1.78 (m, 3H), 1.87–1.92 (m, 1H), 2.02–2.06 (m, 1H), 2.16–2.28 (m, 2H), 2.34–2.41 (m, 1H), 2.63–2.70 (m, 1H), 3.37–3.43 (m, 1H), 3.87–3.93 (m, 1H), 4.45–4.54 (m, 2H), 4.78 (d, *J* = 3.2 Hz, 1H), 5.29 (s, 2H), 5.40 (s, 1H), 6.64 (s, 1H), 7.02–7.03 (m, 2H), 7.64–7.67 (m, 2H). ^13^C-NMR (100 MHz, CDCl_3_) δ (ppm): 162.03, 159.26, 156.18, 142.64, 141.33, 126.52, 123.13, 113.55, 112.67, 112.62, 107.50, 104.21, 102.61, 102.21, 87.95, 80.96, 64.66, 62.45, 52.50, 47.68, 44.27, 37.47, 36.37, 34.56, 30.81, 30.44, 26.14, 24.66, 24.55, 20.36, 13.09. ESI-HRMS [M + Na]^+^: (*m*/*z*) Calcd. for C_31_H_36_F_3_N_3_O_8_Na: 658.2347. Found: 658.2345.

*7-(1-(3-(10β-Dihydroartemisininoxy)propyl)-1H-1,2,3-triazol-4-yl)methoxy-3,4-dimethyl-2H-chromen-2-one* (**13c**): White solid; yield 37%; m.p. 68–70 °C. ^1^H-NMR (400 MHz, CDCl_3_) δ (ppm): 0.93 (d, *J* = 7.6 Hz, 3H), 0.96 (d, *J* = 6.4 Hz, 3H), 1.22–1.40 (m, 3H), 1.43 (s, 3H), 1.46–1.52 (m, 2H), 1.54–1.78 (m, 3H), 1.86–1.95 (m, 1H), 2.01–2.17 (m, 2H), 2.19 (s, 3H), 2.22–2.35 (m, 2H), 2.38 (s, 3H), 2.60–2.69 (m, 1H), 3.36–3.41 (m, 1H), 3.87–3.93 (m, 1H), 4.42–4.53 (m, 2H), 4.77 (d, *J* = 3.6 Hz, 1H), 5.26 (s, 2H), 5.40 (s, 1H), 6.91 (d, *J* = 2.4 Hz, 1H), 6.94 (dd, *J* = 2.4 Hz, *J* = 8.8 Hz, 1H), 7.52 (d, *J* = 8.8 Hz, 1H), 7.65 (s, 1H). ^13^C-NMR (100 MHz, CDCl_3_) δ (ppm): 162.33, 160.02, 153.40, 146.16, 143.22, 125.44, 122.99, 119.31, 114.68, 112.18, 104.18, 102.18, 101.82, 87.93, 80.97, 64.65, 62.28, 52.51, 47.62, 44.27, 37.45, 36.37, 34.56, 30.82, 30.44, 26.15, 24.66, 24.55, 20.36, 15.10, 13.19, 13.09. ESI-HRMS [M + Na]^+^: (*m*/*z*) Calcd. for C_32_H_41_N_3_O_8_Na: 618.2786. Found: 618.2785.

*3-Chloro-7-(1-(3-(10β-Dihydroartemisininoxy)propyl)-1H-1,2,3-triazol-4-yl)methoxy-4-methyl-2H-chromen-2-one* (**13d**): White solid; yield 38%; m.p. 71–73 °C. ^1^H-NMR (400 MHz, CDCl_3_) δ (ppm): 0.93 (d, *J* = 7.6 Hz, 3H), 0.97 (d, *J* = 6.4 Hz, 3H), 1.22–1.29 (m, 2H), 1.32–1.38 (m, 1H), 1.43 (s, 3H), 1.46–1.51 (m, 2H), 1.58–1.82 (m, 3H), 1.87–1.92 (m, 1H), 2.02–2.07 (m, 1H), 2.17–2.25 (m, 2H), 2.33–2.41 (m, 1H), 2.56 (s, 3H), 2.62–2.70 (m, 1H), 3.36–3.42 (m, 1H), 3.87–3.92 (m, 1H), 4.41–4.56 (m, 2H), 4.77 (d, *J* = 3.2 Hz, 1H), 5.27 (s, 2H), 5.40 (s, 1H), 6.95 (d, *J* = 2.0 Hz, 1H), 7.01 (dd, *J* = 2.4 Hz, *J* = 8.8 Hz, 1H), 7.55 (d, *J* = 8.8 Hz, 1H), 7.66 (s, 1H). ^13^C-NMR (100 MHz, CDCl_3_) δ (ppm): 161.04, 157.34, 152.98, 147.86, 142.90, 126.07, 123.06, 118.17, 113.80, 113.08, 104.21, 102.21, 102.09, 87.95, 80.97, 64.65, 62.40, 52.51, 47.65, 44.28, 37.47, 36.38, 34.57, 30.82, 30.45, 26.16, 24.67, 24.56, 20.37, 16.21, 13.10. ESI-HRMS [M + Na]^+^: (*m*/*z*) Calcd. for C_31_H_38_ClN_3_O_8_Na: 638.2240. Found: 638.2240.

*Ethyl 2-oxo-7-(1-(3-(10β-dihydroartemisininoxy)propyl)-1H-1,2,3-triazol-4-yl)methoxy-2H-chromene-3-carboxylate* (**13e**): White solid; yield 30%; m.p. 79–81 °C. ^1^H-NMR (400 MHz, CDCl_3_) δ (ppm): 0.93 (d, *J* = 7.6 Hz, 3H), 0.96 (d, *J* = 5.6 Hz, 3H), 1.22–1.32 (m, 2H), 1.39–1.43 (m, 6H), 1.47–1.56 (m, 2H), 1.61–1.73 (m, 2H), 1.76–1.78 (m, 1H), 1.86–1.93 (m, 1H), 1.97–2.07 (m, 2H), 2.18–2.26 (m, 2H), 2.33–2.41 (m, 1H), 2.62–2.68 (m, 1H), 3.37–3.43 (m, 1H), 3.87–3.92 (m, 1H), 4.40 (q, 2H), 4.45–4.55 (m, 2H), 4.78 (d, *J* = 3.2 Hz, 1H), 5.29 (s, 2H), 5.40 (s, 1H), 6.94 (d, *J* = 2.0 Hz, 1H), 6.99 (dd, *J* = 2.4 Hz, *J* = 8.8 Hz, 1H), 7.53 (d, *J* = 8.8 Hz, 1H), 7.68 (s, 1H), 8.50 (s, 1H). ^13^C-NMR (100 MHz, CDCl_3_) δ (ppm): 163.59, 163.40, 157.32, 157.03, 148.84, 146.18, 130.84, 114.59, 113.84, 112.13, 104.19, 102.13, 101.78, 87.97, 81.01, 80.63, 64.64, 62.26, 61.77, 52.54, 48.49, 44.34, 37.47, 36.39, 34.63, 30.85, 30.14, 29.71, 26.17, 24.68, 20.38, 14.29, 13.16. ESI-HRMS [M + Na]^+^: (*m*/*z*) Calcd. for C_33_H_41_N_3_O_10_Na: 662.2684. Found: 662.2680.

### 3.2. In Vitro Cytotoxicity Study (MTT Assay)

All of the newly-synthesized compounds were screened for *in vitro* cytotoxicity by MTT assay. The exponentially-growing MRC-5, HCT-116, MDA-MB-231, and HT-29 cells were allowed to grow until 80% confluency, the culture medium was discarded, digested with trypsin, separated centrifugally, then fresh culture medium was added and blown gently into single-cell suspension. Then, the cell suspension was seeded into each well of 96-well microculture plates at a concentration of 1.5 × 10^3^–3 × 10^3^ cells/well.

After incubation for 24 h at 37 °C, the four lines of cells were treated with varying concentrations (100, 50, 25, 12.5, and 6.25 μM) of DOX, AAZ, or the derivatives for 96 h. The cells were incubated with 50 μL of 2 mg/mL MTT solution for 4 h in a humidified incubator. The supernatant was discarded, and the media were then replaced with 200 μL of dimethyl sulfoxide to dissolve the purple colored formazan crystals formed in the wells, and their absorbances were measured at 492 nm with a microplate reader (Synergy-HT, BioTek Instruments, Winooski, VT, USA); 200 μL DMSO was set as the blank control.

The anoxia condition was achieved by placing cells in a sealed hypoxia incubator chamber (Catalog Number 27310, Stemcell Technologies, Inc., Vancouver, BC, Canada) filled with 5% CO_2_ and 95% N_2_. For the hypoxia group, the cells were treated with varying concentrations (100, 50, 25, 12.5, and 6.25 μM) of DOX, AAZ, or the derivatives under anoxic condition for 24 h [29], and then moved into nomoxic condition and cultured for additional 72 h.

## 4. Conclusions

In this study, four series of dihydroartemisinin–coumarin hybrids were designed and synthesized, the target compounds were characterized by HRMS, ^1^H-, and ^13^C-NMR. The cytotoxic activities were evaluated by MTT assay against HCT-116, MDA-MB-231, and HT-29 cell lines under normoxic or anoxic conditions, respectively. The target compounds exhibited moderate activity with IC_50_ values in the 0.05–125.40 μM range, and these compounds exhibited better activity against HT-29 cell line under anoxic condition. Under anoxic conditions, cytotoxic activities of most compounds under anoxic conditions displayed one- to 10-fold greater activity than under normoxic condition. We were delighted to find that the cytotoxic activities of compounds **10a**–**e** exhibited better activity against the HT-29 cell line under anoxic conditions compared with the other two cancer cell lines, which indicated that our design of CA IX inhibitors do correspond with its action mode in some degree. On the basis of the preliminary SARs we summarized, further investigations are currently in process.

## Figures and Tables

**Table 1:** IC_50_ values of tested compounds against MRC-5, HCT-116, MDA-MB-231, and HT-29.

Compd.	IC_50_ (μM)									
	MRC-5	HCT-116			MDA-MB-231			HT-29		
	Normoxia	Anoxia	Normoxia	Value ^1^	Anoxia	Normoxia	Value^1^	Anoxia	Normoxia	Value ^1^
**AAZ**	>100	>100	>100	-	>100	>100	-	>100	>100	-
**DOX**	0.34	0.15	0.06	0.39	0.37	0.27	0.73	0.05	0.09	1.74
**10a**	27.98	11.94	10.31	0.86	36.74	52.02	1.42	1.47	12.29	8.35
**10b**	33.56	8.31	6.46	0.78	24.68	28.01	1.13	2.18	10.88	4.99
**10c**	22.31	6.89	6.51	0.95	33.19	32.34	0.97	1.82	20.97	11.50
**10d**	50.72	5.30	4.18	0.79	29.19	61.41	2.10	0.05	17.70	333.96
**10e**	52.47	28.80	51.51	1.79	91.21	>100	1.10	8.69	31.83	3.66
**11a**	15.49	6.02	8.21	1.36	17.82	51.18	2.87	1.22	8.78	7.17
**11b**	43.79	9.33	12.86	1.38	36.90	72.62	1.97	5.06	8.23	1.63
**11c**	60.86	16.56	20.97	1.27	39.75	56.00	1.41	23.20	19.28	0.83
**11d**	>100	6.00	7.68	1.28	22.51	51.60	2.29	1.34	3.90	2.90
**11e**	38.74	28.14	44.14	1.57	50.67	120.72	2.38	12.03	51.09	4.25
**12a**	37.40	18.27	27.44	1.50	47.74	125.40	2.63	10.64	33.05	3.11
**12b**	18.51	24.35	8.47	0.35	14.67	40.05	2.73	2.46	11.34	4.62
**12c**	33.84	12.07	16.01	1.33	33.16	>100	3.02	2.87	17.32	6.04
**12d**	62.33	5.86	15.89	2.71	43.51	>100	2.30	3.63	22.37	6.16
**12e**	69.55	35.67	57.87	1.62	65.06	>100	1.54	20.75	38.96	1.88
**13a**	26.17	9.65	28.49	2.95	36.06	>100	2.77	6.40	27.51	4.30
**13b**	21.40	2.75	20.44	7.43	26.57	>100	3.76	5.31	19.83	3.74
**13c**	27.78	2.13	19.07	8.95	4.17	59.60	14.29	2.80	11.86	4.23
**13d**	31.47	0.43	17.96	41.38	3.62	72.50	20.03	4.68	8.76	1.87
**13e**	27.24	17.60	64.28	3.65	25.94	53.68	2.07	7.09	15.74	2.22

^1^ The values stand for the activity promotion for each cancer cell line. Value = IC_50_(normoxia)/IC_50_(anoxia).
